# Estimating Surgical Blood Loss Volume Using Continuously Monitored Vital Signs

**DOI:** 10.3390/s20226558

**Published:** 2020-11-17

**Authors:** Yang Chen, Chengcheng Hong, Michael R. Pinsky, Ting Ma, Gilles Clermont

**Affiliations:** 1 Department of Electronics and Information Engineering, Harbin Institute of Technology Shenzhen, Shenzhen 518055, China; yichen009@foxmail.com (Y.C.); hongcc2913@163.com (C.H.); 2 Department of Critical Care Medicine, University of Pittsburgh, Pittsburgh, PA 15261, USA; pinsky@pitt.edu (M.R.P.); cler@pitt.edu (G.C.)

**Keywords:** photoplethysmography, arterial blood pressure, blood loss estimation, surgical hemorrhage, machine learning

## Abstract

*Background*: There are currently no effective and accurate blood loss volume (BLV) estimation methods that can be implemented in operating rooms. To improve the accuracy and reliability of BLV estimation and facilitate clinical implementation, we propose a novel estimation method using continuously monitored photoplethysmography (PPG) and invasive arterial blood pressure (ABP). *Methods:* Forty anesthetized York Pigs (31.82 ± 3.52 kg) underwent a controlled hemorrhage at 20 mL/min until shock development was included. Machine-learning-based BLV estimation models were proposed and tested on normalized features derived by vital signs. *Results:* The results showed that the mean ± standard deviation (SD) for estimating BLV against the reference BLV of our proposed random-forest-derived BLV estimation models using PPG and ABP features, as well as the combination of ABP and PPG features, were 11.9 ± 156.2, 6.5 ± 161.5, and 7.0 ± 139.4 mL, respectively. Compared with traditional hematocrit computation formulas (estimation error: 102.1 ± 313.5 mL), our proposed models outperformed by nearly 200 mL in SD. *Conclusion:* This is the first attempt at predicting quantitative BLV from noninvasive measurements. Normalized PPG features are superior to ABP in accurately estimating early-stage BLV, and normalized invasive ABP features could enhance model performance in the event of a massive BLV.

## 1. Introduction

Bleeding is a common surgery complication. Acute blood loss is a life-threatening physiological condition, and the resulting hemorrhagic shock is one of the most common causes of mortality for surgical patients [1,2]. Beyond the assessment of fluid responsiveness, accurate estimation of blood loss volume (BLV) is critical to appropriately resuscitate surgical patients with fluids and blood if necessary. Monitoring and estimating BLV could also be used as a surgical quality indicator to improve the care of surgical patients and reduce patient morbidity, mortality, and health care costs [3,4,5]. Reviewing the entire process of the clinical patient pathway, potential advantages for the estimation of surgical BLV include, but are not limited to, surgical quality review, optimization of anesthesia, transfusion therapy, and surgical management of actively bleeding and coagulopathic patients [4]. 

BLV estimation requires a better solution. There are three main types of BLV estimation methods currently used in clinics: visual estimation, weighing compresses and gauzes, and BLV computation formulas using hematocrit (HCT) values. Among these, visual estimation remains the most frequently used. Physicians have visually estimated BLV based on prior experience. However, this method has proven inaccurate and unreliable, often biased toward overestimating modest blood loss and underestimating large blood loss. This is especially true of inexperienced surgeons [6,7]. Although some improvements, including referring to a nomogram, were proposed for visual estimation, estimation bias remains questionable in clinical implementation [8]. The second most commonly used method, weighing compresses and gauzes, is widely used in surgical operating theaters and delivery rooms. This method performs better than visual estimation but is slow and cumbersome as well as difficult to apply during large and rapid blood loss. The third method is computational formulas using HCT values, based on the principle that HCT would proportionally decrease with the blood volume reduction. BLV can be derived by comparing the current HCT value with its baseline. Based on this principle, several BLV estimation formulas have been proposed to consider the compartmental equilibration corrections necessary to improve estimation performances [9,10,11]. 

The need for reliable methods to estimate BLV approaches is rising, and the use of continuously monitored vital signs has attracted attention. Invasive arterial blood pressure (ABP) is the golden standard for hemorrhage identification in clinics. However, blood pressure (BP) only decreases significantly when a large blood loss has already occurred due to human compensatory responses, especially in young and healthy subjects [12]. Surgical or traumatic hemorrhage often occurs acutely. The poor sensitivity of BP, and particularly the ABP signal, may not be able to yield an accurate estimate of BLV. Other vital signs, including heart rate and blood oxygen saturation, also have low sensitivity and specificity in BLV estimation. 

The noninvasive photoplethysmography (PPG) signal is another potential surrogate for blood volume variations because of its ability to detect local intravascular volume changes. Previous studies have reported that PPG-amplitude-derived features, such as the pleth variability index and amplitude modulations, have the potential for measuring spontaneous blood loss [13,14,15]. A prior attempt was made using the PPG waveform to detect hemorrhage but not for estimating BLV [16]. Among obstacles to using the PPG waveform for tracking hemodynamic changes is the variability that exists among subjects. Simply correcting for moving baselines or amplitudes of the PPG signal does not fully address subject-to-subject variability [17]. Current early hemorrhage detection studies are based on machine learning approaches, where feature normalization by referencing their baseline value could mitigate large subject-to-subject variability [16]. Pinsky et al. reported the efficiency of normalizing features derived by vital signs by referencing their baseline values [18]. It is thus hypothesized that normalized features derived by vital signs could be potential predictors for tracking hemorrhage and BLV. 

In this study, we use an experimental design that simulates surgical hemorrhage in pigs. Critical vital signs ABP and PPG were continuously collected and their waveform features were extracted. Two machine learning methods, the least absolute square shrinkage operation (LASSO) regressor and random forest (RF) regressor, were performed on extracted features to train the BLV estimation model. Three different kinds of models are presented: BLV estimation using solely PPG, solely ABP, and the combination of PPG and ABP. Preliminary results suggest that our proposed models could estimate surgical BLV well on anesthetized pigs and normalized PPG features are superior to ABP in accurately estimating BLV in the early stage of hemorrhage, whereas normalized ABP features could enhance model performance as BLV increases. 

## 2. Methods

### 2.1. Research Subjects and Hemorrhagic Surgery Protocol

**Figure 1:**
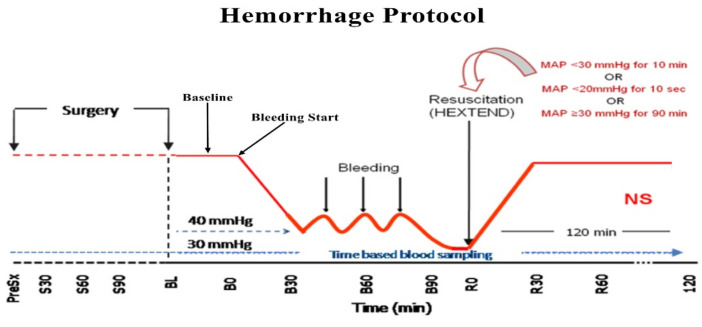
Schematic representation of the hemorrhagic shock protocol. Cited and modified from [19,20] with permission from Elsevier.

Forty Yorkshire Pigs (31.82 ± 3.52 kg) were used. Animals were anesthetized, intubated and ventilated on volume control mode, and instrumented. The PPG signal was monitored in the tail (Masimo, Irvine, CA, USA), and the invasive ABP was monitored (LiDCOplus™, LiDCO Ltd., London, UK) in the femoral artery. After surgical preparation, subjects were observed without any further manipulation to set a baseline for 30 min. Animals were then bled using a roller pump (Masterflex L/S easy-load II pump, Cole-Parmer; Vernon Hills, IL, USA) at a 20 mL/min fixed rate until their mean arterial pressure (MAP) reached 30 mmHg. When subjects were in hemorrhagic shock, fluid resuscitation was performed immediately by femoral vein infusion. Figure 1 depicts a schematic representation of the hemorrhagic shock protocol. Further details of these experiments have been previously presented [19,20]. All experiments were approved by the Institutional Animal Care and Use Committee of the University of Pittsburgh (Protocol Numbers 13061614 and 130923382).

The PPG and ABP signals were collected from the beginning of the baseline period at a sampling rate of 250 Hz. The reference BLV was calculated based on the time elapsed from the start of the bleed and pump bleeding rate according to data in the surgical notes. The initial blood volume of each subject was estimated using their weight. HCT data was measured from blood draws at the baseline, start of bleeding, start of resuscitation, and every 30 min otherwise. Only data accrued prior to resuscitation were used for the current analysis.

### 2.2. Feature Derivation and Normalization

PPG and ABP signals were first reviewed by clinical experts to judge the signal quality and differentiate physiological changes in signal noise induced by vital signs and artifacts based on visual distinction. No signal preprocessing was necessary on major data because of the acceptable signal quality, and other data with artifacts were dropped. Continuous signals were divided into overlapping data frames of a 1 min duration updated every 30 s. Features were computed over these 1 min windows. We selected this window length because 1 min long vital signs contain information to capture significant physiological changes and adequate waveform for feature extraction. 

Graphical features include the amplitude and time domain features extracted from PPG and ABP, and their first derivative waveform has been widely used for tracking hemodynamics [21,22]. Our previous studies also attempted to use features derived from vital signs and machine learning techniques to detect hemorrhage and reported the ability of PPG-waveform-derived features, heart rate, and BP-derived features in reflecting physiological condition changes in subjects suffering from blood loss [18,23,24]. Similar features were extracted as the feature metrics in this study. A detailed explanation of these feature metrics and their graphical diagrams are presented in the Appendix A. For the PPG signal, 18 features were derived. For the ABP signal, 6 features were derived using the ABP-waveform-derived systolic blood pressure (SBP) and diastolic blood pressure (DBP). Six pulse rate (PR)-derived features were also included. The final feature vector of the ABP waveform included 12 features (6 from the ABP signal and 6 from the PR), and the feature vector of the PPG waveform included 24 features (18 from the PPG waveform and 6 from the PR). A feature vector computed over a single 1 min window constituted an observation. 

Two different feature-rescaling approaches were performed to normalize features to the subjects’ corresponding baselines. Given m total subjects, baseline data for the jth subject are designated $VTS_{bsl}^{j}\left(t\right)$. The data in the bleeding period are $VTS_{bld}^{j}\left(t\right)$. Feature vectors extracted from $VTS_{bsl}^{j}\left(t\right)$ and $VTS_{bld}^{j}\left(t\right)$ are $X_{bsl}^{j}=\left\{x_{bsl,1}^{j},x_{bsl,2}^{j},\ldots ,x_{bsl,n}^{j}\right\}$, $X_{bld}^{j}=\left\{x_{bld,1}^{j},x_{bld,2}^{j},\ldots ,x_{bld,n}^{j}\right\}$, respectively. $X_{bsl}^{j}$ is the set of all feature vectors of subject *j*, and $x_{bsl,i}^{j}$ is the ith feature vector in $X_{bsl}^{j}$. 

The first approach is called person-specific feature normalization. For the ith feature vector of subject *j*, we first compute the feature’s mean value in its baseline as the center value then normalize the corresponding subject’s features by dividing the feature by the center value, shown in Equation (1).

(1) $$x_{bldp,i}^{j}=\frac{x_{bld,i}^{j}}{average\left(x_{bsl,i}^{j}\right)}$$

$x_{bldp,i}^{j}$ is the subject-normalized feature vector of $x_{bld,i}^{j}$.

The second feature normalization approach is called group-specific feature normalization. For the ith feature vector of subject *j*, in this approach, the center value is the mean of all the subject’s feature value in their baseline, and the normalized feature $x_{bldg,i}^{j}$ is computed as in (2).

(2) $$x_{bldg,i}^{j}=\frac{x_{bld,i}^{j}}{average\left\{A\right\}}$$

(3) $$A=\left\{x_{bsl,i}^{k}|k\in \;\left(1,m\right),k\neq j\right\}$$

By using these two approaches, features were normalized as the ratio to the baseline; thus, subjects’ differences are addressed. The advantage of group-specific normalization lies in its applicability even in the absence of a personal baseline.

### 2.3. Model Development

We derived models for BLV estimation using a supervised machine learning framework. Two different machine learning methods, a RF regressor and a LASSO regressor, were trained in this study. RF is an ensemble method combining multiple decision trees to avoid overfitting, and LASSO is a linear method that performs both regression and feature selection with L1 regularization. Data used for model training were observations from both baseline data and bleeding data. The label of each observation was 0 (for all baseline feature vectors) or the computed BLV at that time from feature vectors computed from bleeding data. We then used the trained models to estimate the BLV. The input of the trained models was the observations and the output was the estimated BLV at that time. To avoid model overfitting, we conducted our experiments using a leave-one-animal-out cross-validation (CV) protocol. Observations from one subject would be designated as the test set, while observations from the other thirty-nine subjects were the training set. Our models were developed using the *scikit-learn* toolbox in the Python 3.6 environment [25]. For the LASSO model, the *alpha* parameter was set as the hyperparameter for optimization. For the RF model, we optimized hyperparameters *n_estimators* and *max_depth* by grid search. 

We finally proposed six different models on different feature sets: (1) a model using PPG features and personal-specific feature normalization (PPG Personal Model); (2) a model using PPG features and group-specific feature normalization (PPG Group Model); (3) a model using ABP features and personal-specific feature normalization (ABP personal Model); (4) a model using ABP features and group-specific feature normalization (ABP Group Model); (5) a model using the combination of ABP and PPG features and personal-specific feature normalization (ABP&PPG Personal Model); (6) a model using the combination of ABP and PPG features and group-specific feature normalization (ABP&PPG Group Model). Twelve features were used in the ABP model, 24 features were used in the PPG model, and 30 features were used in the ABP&PPG model. It is noted that, for personal models, features were normalized using personal-specific methods before model training and testing. For the group models, for each CV epoch, features in the training set were first normalized using the group-specific method, and the same parameters obtained from the training set were performed on features in the test set for normalization. The difference in mean and standard deviation (SD) between actual and predicted BLV on the observations from the hold-out subject at all available time points was used as the model’s evaluation metric. 

We also compared our model predictions to three traditional HCT-based blood loss estimation formulas proposed by Ward et al., Bourke et al., and Gross et al. when we obtained HCT values. These formulas are provided by the following equations [9,10,11]:

(4) $$Blood\;loss\;=EBV\;\times ln\;ln\;\frac{Hct_{f}}{Hct_{i}}$$

(5) $$Blood\;loss=EBV\;\times \;\left(Hct_{i}-Hct_{f}\right)\;\times \;\left(3-\;Hct_{mean}\right)\;$$

(6) $$Blood\;loss=EBV\;\times \;\frac{Hct_{i}-\;Hct_{f}}{Hct_{mean}}$$

EBV is the subjects’ estimated initial blood volume, $Hct_{i}$ is the HCT at baseline, $Hct_{f}$ is the HCT in the bleeding stage, and $Hct_{mean}$ is the mean HCT between the baseline and the bleeding stage. Finally, features’ explanatory contribution to the BLV estimation models were also investigated using the Gini-impurity index for the RF model and the size of coefficients of features for the LASSO Model prediction equation [26]. Because coefficients of the LASSO Model may be negative, we computed the mean absolute values of the feature’s importance among different CV folds and ranked them. 

## 3. Results

### 3.1. Characteristics of Research Subjects 

The mean initial blood volume in total was 2160 ± 237 mL, with a maximum of 2645 mL and a minimum of 1727 mL. The final blood loss volume was 1031.5 ± 377.8 mL. The animals’ total blood loss was 47.7 ± 16.3% of their estimated initial blood volume. Maximum and minimum BLV were 1730 and 710 mL. According to the surgical notes, the longest and shortest baseline periods among all subjects were 31 and 18 min, where the longest and shortest bleeding periods were 185 and 106 min. There were 11,032 observation windows, with 1823 observations in the baseline period and 9209 observations in the bleeding period. A total of 366 observations (3.3%) were dropped because of missing data or artifacts. The final dataset includes 10,666 observations, with an average number of 266.7 observations per subject. Since the dimension of our models’ feature metrics is 12–30, this number of observations is adequate to precisely determine the actual performance of our proposed models [27]. 

### 3.2. Model Performance of Blood Loss Estimation

The hyperparameters of our proposed models are listed below. For all LASSO-derived models, the *alpha* parameter was set as 0.05 to balance the estimation error and model complexity. For RF-derived models using PPG features, *n_estimators* = *34* and *max_depth* = *12*. For RF models using ABP features, *n_estimators* = *15* and *max_depth* = *8*. For RF models using ABP&PPG model, *n_estimators* = *43* and *max_depth* = *19*.

The PPG personal model using the RF regressor achieves an overall estimation error of 11.9 ± 156.2 mL (mean ± SD), and the performance of the ABP personal model using RF is 6.5 ± 161.5 mL. However, if PPG and ABP are combined, the estimation error decreases to 7.0 ± 139.4 mL. We also developed models using non-normalized features. However, because of their extremely terrible performance, we will not discuss them further.

**Figure 2:**
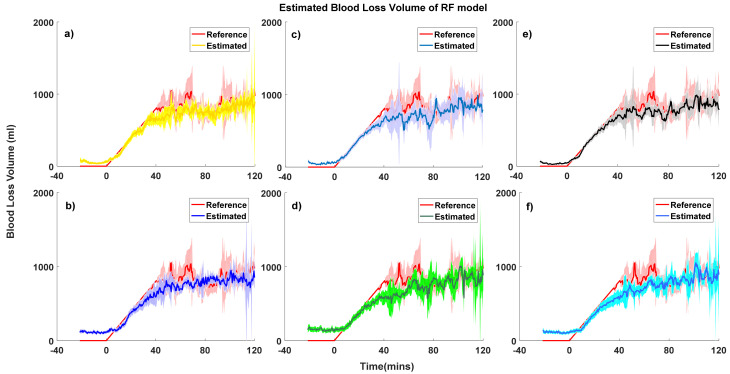
Estimated and reference blood loss curves of the random forest (RF) model with 95% confidence intervals. (**a**) Arterial blood pressure (ABP) personal model, (**b**) ABP grouped model, (**c**) photoplethysmography (PPG) personal model, (**d**) PPG grouped model, (**e**) arterial blood pressure and photoplethysmography (ABP&PPG) personal model, and (**f**) ABP&PPG grouped model.

**Figure 3:**
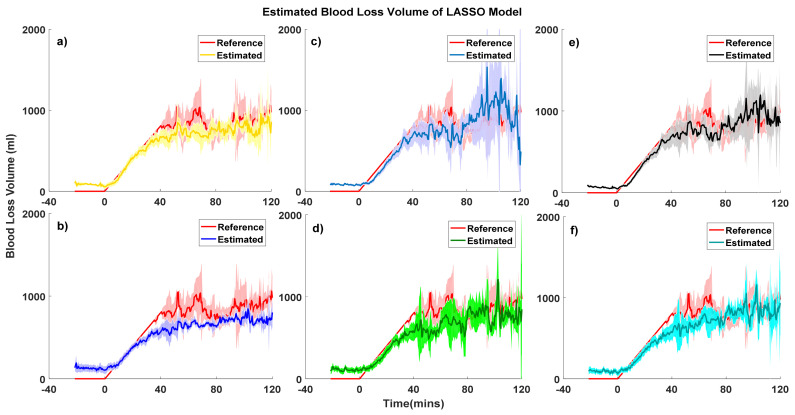
Estimated and reference blood loss curves of the least absolute square shrinkage operation (LASSO) model with 95% confidence intervals. (**a**) ABP personal model, (**b**) ABP grouped model, (**c**) PPG personal model, (**d**) PPG grouped model, (**e**) ABP&PPG personal model, and (**f**) ABP&PPG grouped model.

To further investigate the proposed models’ performance at different degrees of BLV severity, we classified our data into the following five classes: no bleeding, a loss of <15% of the blood volume, a loss of 15–30% of the blood volume, a loss of 30–45% blood volume, and a loss of >45% blood volume [12]. Details of the models’ performance in different blood loss severities are summarized in Table 1. Blood loss volume estimation curves of RF models and LASSO models are plotted in Figure 2 and Figure 3, respectively.

**Figure 4:**
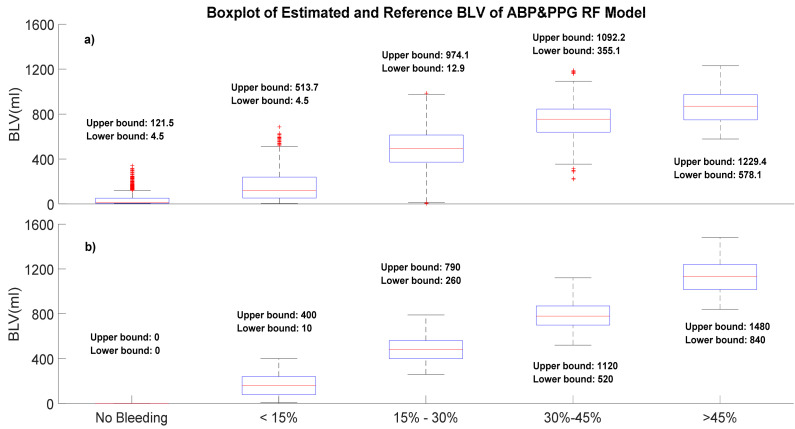
Boxplot of estimated and reference BLV of the RF-derived ABP&PPG person-specific model. (**a**) Estimated BLV; (**b**) reference BLV.

Generally, models developed using RF tended to have slightly higher biases and higher precision than LASSO models. As expected, the performance of personal models showed less bias and comparable precision compared to the group-normalized models, but the difference was not clinically significant. One interesting finding is, in the RF-derived model, when subjects suffer around 15% to 30% blood loss, the bias between the estimated BLV and reference BLV is much higher than the other bleeding classes. To further analyze this finding, we devised a boxplot of the estimated and reference BLV of the RF-derived ABP&PPG person-specific model, presented in Figure 4. A significant bias is investigated when subjects are in the 15% to 30% blood loss classes. The reference BLV is from 260 to 790 mL, but the estimated BLV is from 12.9 to 974.1 mL. This bias may be the potential cause of the large variation of the estimation error.

Another interesting finding is that models using only the PPG signal had comparable performance to models including ABP in the early stage of hemorrhage (BLV < 30%). Models uniformly underpredicted actual blood loss when BLV > 30%. 

Model prediction at specific time points when HCT values were obtained were also compared to the three HCT-based formulas, as summarized in Table 2. The mean errors of these three HCT-based formulas in our data are 134.7 ± 343.4, 102.1 ± 313.5, and 131.2 ± 341.4 mL. Performances of traditional HCT-based blood loss estimation formulas are significantly worse than our proposed method, more apparent when BLV < 30%. Though HCT values do decrease with bleeding, they do not vary proportionally with the BLV, which might be the main reason why the traditional HCT-based formulas tended to underestimate BLV. 

### 3.3. Comparisons of Feature Importance among Models

The relative importance of features explaining model predictions was examined and the results are presented. Table 3 and Table 4 show the first 22 average positions in the ranking of the LASSO and RF-derived models, respectively. 

The PPG signal peak–foot amplitude is the highest-ranked feature in the LASSO models using the PPG signal, while PIR is the second highest-ranked feature (Appendix A). These two features are PPG-amplitude-related features. In addition to PPG-amplitude-related features, the time interval between PPG characteristic points and area under specific PPG waveforms play an important role in BLV estimation models. When we examined ABP related features, the mean values of SBP, DBP, and PP were the most predictive. Feature ranks between personal models and group models are also different. For group models, features related to the variations of vital signs ranked higher than it in the personal model.

Table 4 presents the feature ranking in RF models. The ranking is generally consistent with that provided with the LASSO-derived models, with PPG amplitudes carrying more importance in PPG-based models. Interestingly, the mean DBP value is the first rank feature in the majority of these models, while traditional measures of functional hemodynamics, such as pulse pressure and pulse pressure variation, did not carry much predictive value. 

**Figure 5:**
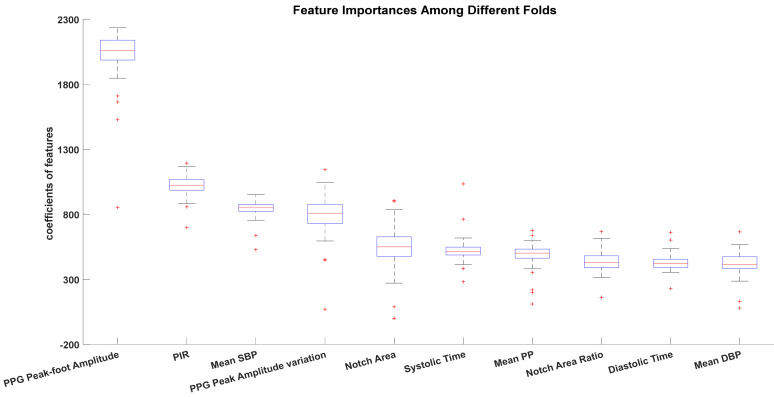
Features’ importance variation among different folds of the first 10 importance features of the LASSO-derived person-specific model using ABP&PPG features.

For LASSO-derived models, coefficients of useless features were set as 0. We found that, in our study, there were no features with 0 coefficients in the personal model using ABP features during each CV fold. Regarding the personal model using PPG features, an average of 2.4 ± 0.5 features were set as 0 coefficients in each CV folds. The personal models using ABP&PPG features have 3.1 ± 0.6 features with 0 coefficients in each CV folds. No feature was with 0 coefficients in all CV folds in all models. Features’ importance of the ABP&PPG personal models among different CV folds were also investigated, and their boxplots are shown in Figure 5 for the LASSO model. There is consistency in the relative importance of coefficient across validation folds and a mix of PPG- and ABP-derived features. 

## 4. Discussion

We proposed novel models toward accurate BLV estimation for surgical hemorrhage using continuously monitored vital signs and a supervised machine learning framework. Models built using only features from noninvasive PPG waveforms showed on average minimal bias and good performance (11.9 ± 156.2 mL estimation error), yet tended to significantly underestimate blood loss exceeding 30% of the initial blood volume. This performance was comparable to models using only the invasive ABP waveform (6.5 ± 161.5 mL). However, if the ABP and PPG signals were combined, model performance was significantly improved (7.0 ± 139.4 mL estimation error), although this improvement is likely not clinically significant. We also found that PPG models perform as well as invasive ABP models in the early stage of bleeding, but adding ABP improves performance with large bleeds. 

This represents, to our knowledge, the first attempt at predicting quantitative blood loss from noninvasive measurements. The PPG waveform has previously been used to evaluate fluid responsiveness, where a pulse variability index of 15% is associated with fluid responsiveness in subjects receiving positive-pressure ventilation in sinus rhythm [15,28]. Most patients are not monitored invasively using an indwelling arterial catheter, and exploring the conditions under which data from the PPG waveform might provide useful information continues to be warranted. Without some form of signal normalization, the PPG waveform could not predict blood loss. Because of the baseline variability of PPG waveforms across subjects, the mean values of PPG-derived features varied largely among subjects (data not shown). Group-based normalization improves the situation substantially and opens the possibility of deploying such algorithms to situations where a person-specific baseline is not available. However, group-based normalization was also associated with significant bias, as expected. Indeed, any given subject may be quite distinctive. Person-based normalization improves the situation substantially in that bias is significantly reduced. However, person-specific normalization must be interpreted with the caveat that the baseline is assumed to represent a period of stability. We have not specifically explored situations where this assumption is relaxed and the baseline simply represents a group of observations from some time in the past. Given this experimental setup, a blood loss model would possibly identify ongoing blood loss from an unknown initial state. This remains to be explored.

Unlike noninvasive PPG, invasive ABP remains the clinical gold standard for monitoring hemorrhage. In our study, both ABP group-specific models and ABP person-specific models achieved a reasonable bias and estimation error in estimating BLV. We also found there is less than a 20% performance improvement for ABP models using person-specific normalization compared to group-specific normalization. We note that, in our experimental setting, absolute values of ABP were used to define shock (systolic ABP < 30 mmHg) rather than tissue-perfusion-based measurements. Thus, there could be some bias favoring ABP models. We also note that we used the continuous invasive ABP waveform from an arterial line rather than a noninvasively continuously monitored BP, such as can be obtained commercially, yet rarely available, technology. It remains unknown whether our findings regarding ABP models would translate to noninvasive BP monitoring.

An interesting finding of our study is that PPG models performed well in the early stage of bleeding. We believe this may be related to the autonomic compensatory response, which attempts to preserve tissue perfusion and blood pressure early in the bleeding as cardiac output and intravascular volume decrease. We indeed consider pulse rate a significant predictor of BLV, as expected. However, blood flow and blood volume are decreased in proportion to BLV. Thus, we expect an impact on the PPG waveforms. As mentioned below, it is possible that more exhaustive featurization of the arterial waveform could also capture the early physiologic response.

Analysis of the leading predictive features may help provide construct validity to the presented models. There are significant differences in the high-ranking features between LA models and RF models. For the LA models, the most predictive features are PPG-amplitude-related features. It is known that changes in PPG amplitude correlate to changes in the circulatory blood volume, and the area under the PPG waveform positively correlates to the stroke volume [29]. It is interesting to note that the pulse variability index, documented as a good predictor of fluid responsiveness in subjects on positive-pressure ventilation, is only highly ranked in the RF model. It is also noted that the geometric features of the PPG signals are not generally ranked highly in their ability to predict BLV. Thus, the LASSO model could discriminate well between different hemorrhagic situations. Regarding the RF-derived model, nonlinear BP-related features, such as DBP, SBP, and PP, are more important. Although its variation in the hemorrhagic procedures is not linear and significant, the RF regressor could also select it because it is informative.

Our proposed models achieve an acceptable estimation error for the quantitative BLV estimation. It is also investigated that our estimation results reveal a large variation. This large variation potentially demonstrates that, in our study, the model’s performances varied largely among different subjects and different blood loss classes. The results presented in Figure 4 also demonstrate the larger bias between estimated BLV and reference BLV when the subjects are in a 15–30% blood loss class. We believe this large variation of estimation error is reasonable. Different subjects may have different responses when suffering from hemorrhage. For example, a variation in vital signs is not significant for stronger subjects, and our trained models cannot accurately quantify these tiny physiological changes. Other factors, such as artifacts induced by blood draw to collect a blood sample and saturation changes in vital signs when huge bleeding occurs, may also impact the estimation results, causing this large variation.

Our study subjects suffered multiple bleedings until the development of hemorrhagic shock in our experimental protocol. Between each bleeding, a long resting time was performed for each subject to stabilize their vital signs. During these long resting times, the autonomic compensatory response works, blood volume from the capillary is refilled to the aorta and peripheral arteries, and BP and blood volume are recovered. It is reasonable to assume that autonomic compensatory responses would potentially influence the estimation error and cause a larger bias between estimated BLV and reference BLV in certain blood loss classes. 

The main limitation of our study lies in our tightly controlled experimental design, where signals were obtained from anesthetized animals subjected to a strict bleeding protocol. Whether this translates to uncontrolled human settings remains to be explored, as the physiological responses of pigs and humans may be significantly different. Our models slightly overestimate BLV in the early stage of hemorrhage but significantly underestimate BLV with large blood loss. Presumably, such larger blood losses would be clinically apparent. We did not use as extensive a featurization of the ABP signal as we did for PPG, thus potentially biasing the model in favor of PPG. The requirement for baseline normalization limits the potential generalizability of the models to situations where such a baseline is available, such as quantification of surgical or peripartum bleeding. Indeed, peripartum hemorrhage could represent an important use case for models based on noninvasive signals since these patients are rarely instrumented and the majority of hemorrhages occur in low-risk patients. The reasonable performance of models using a population-derived baseline introduces the possibility that such models could be deployed to other use cases where a baseline is not readily available, such as traumatic hemorrhage. Finally, models were developed on observations prior to interventions, such as vasopressors or fluid resuscitation. Model predictions should be evaluated in observations that extend to the resuscitation phase of our experimental design. Another limitation is that only 40 subjects were involved in our study, and there are little data for model development and model validation separately. The result we presented is an optimized value of each CV epoch, leading to an overestimation of our models’ performance. The machine learning models we used were LASSO and RF, which are basic and simple, and the models’ performances heavily rely on the features’ metrics we extracted. Advanced machine learning approaches, such as the deep learning network, could be considered in future work for their ability to automatically perform feature extraction. Further work is currently in progress to address such limitations, devise a more sophisticated machine learning scenario that contains feature engineering and model development work, and potentially extend the ability to quantify blood loss to a wider range of clinical situations beyond controlled surgical hemorrhage.

## 5. Conclusions

We proposed a novel BLV estimation model for surgical hemorrhage using continuously monitored vital signs by referencing the baseline and preoperative signals. Preliminary results suggest that our model could achieve a much smaller estimation error than traditional clinical HCT-based formulas. We also found that a model using the noninvasive PPG signal could be superior to a model derived from the invasive ABP signal in estimating blood loss in the early stage of bleeding. The ABP signal is more predictive of large blood loss. Our results should be confirmed in a human cohort at a high risk of operative hemorrhage. A PPG-derived model could indicate the need for more invasive monitoring and potentially guide intraoperative fluid resuscitation and blood transfusion.

## Figures and Tables

**Table 1:** Models’ performances in different blood loss severities.

Model Performance	PPG-Personal Model-RF (mL)	PPG-Grouped Model-RF (mL)	ABP-Personal Model-RF (mL)	ABP-Grouped Model-RF (mL)	PPG&ABP-Personal Model-RF (mL)	PPG&ABP-Grouped Model-RF (mL)
Overall	11.9 ± 156.2	−21.9 ± 210.6	6.5 ± 161.5	−9.15 ± 193.3	7.0 ± 139.4	−7.6 ± 187.1
No bleeding	−51.8 ± 64.8	−152.9 ± 138.4	−53.6 ± 76.3	−115.7 ± 87.9	−38.0 ± 50.6	−109.6 ± 94.1
Loss of <15%	1.8 ± 123.2	−26.2 ± 171.2	10.5 ± 140.5	3.8 ± 140.3	9.1 ± 103.4	−2.5 ± 144.3
Loss of 15–30%	22.2 ± 158.7	29.7 ± 203.5	−3.8 ± 180.1	21.5 ± 256.2	−1.4 ± 160.3	44.1 ± 191.4
Loss of 30–45%	80.4 ± 193.3	91.3 ± 215.7	57.4 ± 183.1	54.2 ± 215.2	47.3 ± 167.9	64.9 ± 203.8
Loss of >45%	281.8 ± 263.5	320.7 ± 244.9	348.6 ± 198.6	352.2 ± 201.1	264.0 ± 228.0	306.0 ± 214.8
**Model Performance**	**PPG-Personal Model-LASSO (mL)**	**PPG-Grouped Model-LASSO (mL)**	**ABP-Personal Model-LASSO (mL)**	**ABP-Grouped Model-LASSO (mL)**	**PPG&ABP-Personal Model-LASSO (mL)**	**PPG&ABP-Grouped Model-LASSO (mL)**
Overall	−3.6 ± 211.1	9.0 ± 226.8	1.32 ± 180.1	2.9 ± 226.3	3.2 ± 163.2	15.7 ± 208.7
No bleeding	−82.8 ± 58.6	−101.9 ± 149.5	−83.39 ± 85.8	−131.9 ± 195.1	−65.7 ± 53.6	−88.8 ± 138.6
Loss of <15%	35.0 ± 109.1	8.2 ± 178.5	12.13 ± 147.1	−17.3 ± 175.0	38.2 ± 93.4	15.1 ± 162.7
Loss of 15–30%	53.8 ± 191.0	66.7 ± 207.0	−5.71 ± 195.7	38.7 ± 159.2	28.7 ± 175.7	45.7 ± 207.1
Loss of 30–45%	−17.8 ± 360.8	102.4 ± 264.5	77.13 ± 172.9	138.5 ± 169.7	1.1 ± 237.3	78.5 ± 227.9
Loss of >45%	121.2 ± 470.4	379.1 ± 367.3	393.32 ± 204.0	474.3 ± 181.4	209.3 ± 322.5	348.9 ± 277.9

**Table 2:** Hematocrit (HCT) values and variations among different experiment stages.

Blood Loss Stage	HCT Value (%)	HCT Variation (%)	Estimated BLV Error-Ward’s (mL)	Estimated BLV Error-Bourke’s (mL)	Estimated BLV Error-Gross’s (mL)
Baseline	30.8 ± 6.0	\	\	\	\
Blood loss < 30%	28.4 ± 4.8	−9.6 ± 7.1	238.7 ± 295.6	188.8 ± 286.7	236.4 ± 293.5
Blood loss > 30%	28.3 ± 5.7	−3.9 ± 13.8	98.9 ± 390.1	70.9 ± 354.5	93.2 ± 388.0
Over all	\	\	134.7 ± 343.4	102.1 ± 313.5	131.2 ± 341.4

**Table 3:** Features’ importance ranking in the LASSO models.

Features	PPG Personal	PPG Grouped	ABP Personal	ABP Grouped	PPG&ABP Personal	PPG&ABP Grouped
Mean Pulse Rate	5	9	5	7	13	5
Min Pulse Rate	20	5	6	9	19	7
PPG peak Amplitude	9	11	\	\	20	9
PPG peak Amplitude Variation	11	15	\	\	4	18
PPG peak–foot amplitude	1	1	\	\	1	10
PPG peak–foot amplitude variation	10	4	\	\	26	3
Slope Transit Time	12	22	\	\	12	19
Systolic Time	4	6	\	\	6	2
Systolic Area	14	8	\	\	16	13
Systolic Area Ratio	15	10	\	\	15	14
Notch Area	8	14	\	\	5	29
Notch Area Ratio	6	25	\	\	8	30
Diastolic time	3	7	\	\	9	6
Diastolic Area	21	3	\	\	25	16
Diastolic area ratio	7	13	\	\	17	27
PIR	2	2	\	\	2	4
Mean SBP	\	\	1	2	3	8
Mean DBP	\	\	2	1	10	1
Mean PP	\	\	4	3	7	11
Mean SBP variation	\	\	3	5	14	17
Mean DBP Variation	\	\	6	6	18	22
Mean PP Variation	\	\	7	4	19	12

**Table 4:** Features’ importance ranking in RF models.

Features	PPG Personal	PPG Grouped	ABP Personal	ABP Grouped	PPG&ABP Personal	PPG&ABP Grouped
Mean Pulse Rate	6	9	5	6	3	13
Min Pulse Rate	16	17	7	8	13	18
PPG Peak Amplitude	2	6	\	\	14	8
PPG Peak Amplitude Variation	3	5	\	\	12	4
PPG Peak–Foot Amplitude	1	1	\	\	7	2
PPG Peak–Foot Amplitude Variation	5	3	\	\	22	11
Slope Transit Time	8	15	\	\	23	12
Systolic Time	9	2	\	\	6	10
Systolic Area	11	6	\	\	11	9
Systolic Area Ratio	15	10	\	\	18	6
Notch Area	17	7	\	\	8	16
Notch Area Ratio	10	4	\	\	16	14
Diastolic Time	14	11	\	\	9	19
Diastolic Area	4	5	\	\	5	15
Diastolic Area Ratio	20	12	\	\	15	29
PIR	7	8	\	\	10	5
Mean SBP	\	\	3	2	2	3
Mean DBP	\	\	1	1	1	1
Mean PP	\	\	4	3	4	7
Mean SBP Variation	\	\	2	4	27	26
Mean DBP Variation	\	\	8	10	28	17
Mean PP Variation	\	\	6	5	30	24

**Figure A1:**
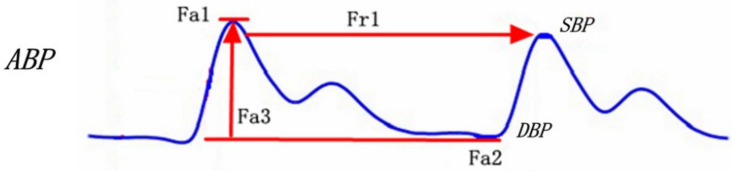
Diagrams of ABP and pulse derived features.

**Figure A2:**
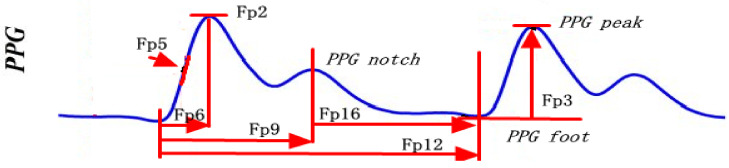
Diagrams of PPG-derived features.

## Appendix A

We present the details of our feature metric in the Appendix A. Table A1 introduces the ABP- and PR-derived features, and Table A2 introduces the PPG-derived features. Diagrams of features are explained in Figure A1 and Figure A2.

**Table A1:** Blood pressure (BP)- and PR-derived features.

Feature Number	Feature Name	Feature Interpretation
Fr1	Mean pulse rate	Mean pulse rate
Fr2	Std pulse rate	Standard deviation of pulse rate
Fr3	RMSDD	Square root of the mean squared differences of pulse intervals
Fr4	PRV COSEN	Coefficient of sample entropy
Fr5	Max PR	Maximum pulse rate in the window
Fr6	Min PR	Minimum pulse rate in the window
Fa1	Mean SBP	Mean SBP in the window
Fa2	Mean DBP	Mean DBP in the window
Fa3	Mean PP	Mean PP in the window
Fa4	Mean SBPV	Mean SBP variation in the window
Fa5	Mean DBPV	Mean DBP variation in the window
Fa6	Mean PPV	Mean PP variation in the window

**Table A2:** PPG-waveform-derived features.

Feature Number	Feature Name	Feature Interpretation
Fp1	PPG Amplitude Variation	Variation of PPG peak amplitude
Fp2	PPG Amplitude	PPG peak amplitude
Fp3	PPG peak–foot amplitude	PPG peak amplitude–PPG foot amplitude
Fp4	PPG Peak–Foot Amplitude Variation	Variation of (PPG peak amplitude–PPG foot amplitude)
Fp5	Slope Transit Time	Slope transit time of PPG
Fp6	Systolic Time	time interval from PPG foot to PPG peak
Fp7	Systolic Area	Area under the systolic wave
Fp8	Systolic Area Ratio	Area under the systolic wave/total beat area
Fp9	Notch Time	Time interval from PPG peak to PPG notch
Fp10	Notch Area	Area under curve from PPG peak to PPG notch
Fp11	Notch Area Ratio	Area under curve from PPG peak to PPG notch/total beat area
Fp12	Diastolic Time	time interval from peak to foot
Fp13	Diastolic Area	Area under diastolic wave
Fp14	Diastolic Area Ratio	Area under the diastolic wave/total beat area
Fp15	PPG Intensity Ratio	PPG peak amplitude/(PPG foot amplitude
Fp16	Notch-Foot Time	Time interval from PPG notch to PPG foot
Fp17	Notch-Foot Area	Area under curve from PPG notch to PPG foot
Fp18	Notch-Foot Area Ratio	Area under curve from PPG notch to PPG foot/total beat area

## References

[1] Asehnoune K., Balogh Z., Citerio G., Cap A., Billiar T., Stocchetti N., Cohen M.J., Pelosi P., Curry N., Gaarder C. (2017). The research agenda for trauma critical care. Intensive Care Med..

[2] Tisherman S.A., Stein D.M. (2018). ICU Management of Trauma Patients. Crit. Care Med..

[3] Clevenger B., Mallett S.V., Klein A.A., Richards T. (2015). Patient blood management to reduce surgical risk. Br. J. Surg..

[4] Goodnough L.T., Shander A. (2012). Patient Blood Management. Anesthesiology.

[5] Shander A., Bracey A.W., Goodnough L.T., Gross I., Hassan N.E., Ozawa S., Marques M.B. (2016). Patient Blood Management as Standard of Care. Anesth. Analg..

[6] Jaramillo S., Montane-Muntane M., Capitan D., Aguilar F., Vilaseca A., Blasi A., Navarro-Ripoll R. (2019). Agreement of surgical blood loss estimation methods. Transfusion.

[7] Rothermel L.D., Lipman J.M. (2016). Estimation of blood loss is inaccurate and unreliable. Surgery.

[8] Yeung C.Y., Yim W.W., Chan S.Y., Lo R.S.L., Leung L.Y., Hung K.K.C., Graham C.A. (2017). Improvement of blood loss volume estimation by paramedics using a pictorial nomogram: A developmental study. Injury.

[9] Bourke D.L., Smith T.C. (1974). Estimating Allowable Hemodilution. Anesthesiology.

[10] Ward C.F., Meathe E.A., Benumof J.L., Trousdale F. (1980). A Computer Nomogram for Blood Loss Replacement. Anesthesiology.

[11] Lopez-Picado A., Albinarrate A., Barrachina B. (2017). Determination of Perioperative Blood Loss: Accuracy or Approximation?. Anesth. Analg..

[12] Marino P.L. (2014). The ICU Book.

[13] Selvaraj N., Scully C.G., Shelley K.H., Silverman D.G., Chon K.H. Early Detection of Spontaneous Blood Loss using Amplitude Modulation of Photoplethysmogram. Proceedings of the 2011 Annual International Conference of the IEEE Engineering in Medicine and Biology Society.

[14] Shamir M., Eidelman L.A., Floman Y., Kaplan L., Pizov R. (1999). Pulse oximetry plethysmographic waveform during changes in blood volume. Br. J. Anaesth..

[15] Cannesson M., Attof Y., Rosamel P., Desebbe O., Joseph P., Metton O., Bastien O., Lehot J.J. (2007). Respiratory variations in pulse oximetry plethysmographic waveform amplitude to predict fluid responsiveness in the operating room. Anesthesiology.

[16] Scully C.G., Selvaraj N., Romberg F.W., Wardhan R., Ryan J., Florian J.P., Silverman D.G., Shelley K.H., Chon K.H. (2012). Using Time-Frequency Analysis of the Photoplethysmographic Waveform to Detect the Withdrawal of 900 mL of Blood. Anesth. Analg..

[17] Cejnar M., Kobler H., Hunyor S.N. (1993). Quantitative Photoplethysmography—Lambert-Beer Law or Inverse Function Incorporating Light Scatter. J. Biomed. Eng..

[18] Pinsky M.R., Wertz A., Clermont G., Dubrawski A. (2020). Parsimony of Hemodynamic Monitoring Data Sufficient for the Detection of Hemorrhage. Anesth. Analg..

[19] Gómez H., Mesquida J., Hermus L., Polanco P., Kim H.K., Zenker S., Torres A., Namas R., Vodovotz Y., Clermont G. (2012). Physiologic responses to severe hemorrhagic shock and the genesis of cardiovascular collapse: Can irreversibility be anticipated?. J. Surg. Res..

[20] Gomez H., Kautza B., Escobar D., Nassour I., Luciano J., Botero A.M., Gordon L., Martinez S., Holder A., Ogundele O. (2015). Inhaled Carbon Monoxide Protects against the Development of Shock and Mitochondrial Injury following Hemorrhage and Resuscitation. PLoS ONE.

[21] Miao F., Fu N., Zhang Y., Ding X., Hong X., He Q., Li Y. (2017). A Novel Continuous Blood Pressure Estimation Approach Based on Data Mining Techniques. IEEE J. Biomed. Health Inform..

[22] Miao F., Liu Z., Liu J., Wen B., He Q., Li Y. (2020). Multi-Sensor Fusion Approach for Cuff-Less Blood Pressure Measurement. IEEE J. Biomed. Health Inform..

[23] Chen Y., Yoon J.H., Pinsky M.R., Ma T., Clermont G. (2020). Development of hemorrhage identification model using non-invasive vital signs. Physiol. Meas..

[24] Wertz A., Holder A.L., Guillame-Bert M., Clermont G., Dubrawski A., Pinsky M.R. (2019). Increasing Cardiovascular Data Sampling Frequency and Referencing It to Baseline Improve Hemorrhage Detection. Crit. Care Explor..

[25] Pedregos F., Varoquau G., Gramfor A., Miche V., Thirio B., Grisel O., Blonde M., Prettenhofer P., Weiss R., Dubour V. (2011). Scikit-learn: Machine Learning in Python. J. Mach. Learn. Res..

[26] Breiman L. (2001). Random Forests. J. Mach. Learn..

[27] Beleites C., Neugebauer U., Bocklitz T., Krafft C., Popp J. (2013). Sample size planning for classification models. Anal. Chim. Acta.

[28] Nilsson L.M. (2013). Respiration signals from photoplethysmography. Anesth. Analg..

[29] John A. (2007). Photoplethysmography and its application in clinical physiological measurement. Physiol. Meas..

